# Therapeutic Effects of Autologous Tumor-Derived Nanovesicles on Melanoma Growth and Metastasis

**DOI:** 10.1371/journal.pone.0033330

**Published:** 2012-03-15

**Authors:** Eun-Young Lee, Kyong-Su Park, Yae Jin Yoon, Jaewook Lee, Hyung-Geun Moon, Su Chul Jang, Kyoung-Ho Choi, Yoon-Keun Kim, Yong Song Gho

**Affiliations:** 1 Department of Life Science and Division of Molecular and Life Sciences, Pohang University of Science and Technology (POSTECH), Pohang, Republic of Korea; 2 Department of Emergency Medicine, College of Medicine, The Catholic University of Korea, Seoul, Republic of Korea; Duke University Medical Center, United States of America

## Abstract

Cancer vaccines with optimal tumor-associated antigens show promise for anti-tumor immunotherapy. Recently, nano-sized vesicles, such as exosomes derived from tumors, were suggested as potential antigen candidates, although the total yield of exosomes is not sufficient for clinical applications. In the present study, we developed a new vaccine strategy based on nano-sized vesicles derived from primary autologous tumors. Through homogenization and sonication of tumor tissues, we achieved high yields of vesicle-bound antigens. These nanovesicles were enriched with antigenic membrane targets but lacked nuclear autoantigens. Furthermore, these nanovesicles together with adjuvant activated dendritic cells *in vitro*, and induced effective anti-tumor immune responses in both primary and metastatic melanoma mouse models. Therefore, autologous tumor-derived nanovesicles may represent a novel source of antigens with high-level immunogenicity for use in acellular vaccines without compromising safety. Our strategy is cost-effective and can be applied to patient-specific cancer therapeutic vaccination.

## Introduction

Studies have demonstrated that antigens expressed on the surface of cancer cells are recognized by the host immune systems [1]. This concept has been used to develop cancer vaccines, which involve active cancer immunotherapy that stimulates the patient's own immune system to destroy the tumor cells. Anti-tumor immunotherapy has the potential to remove not only growing tumors but also metastasized tumors with fewer adverse effects than chemotherapy or radiation therapy [2]. However, the success of cancer vaccines is often limited by a failure of the immune system to recognize the cancer cells [3]. Since most cancer types (∼80%), including melanoma, develop *via* alterations to normal host cells rather than infection, the optimal cancer vaccine should enhance tumor-specific immune responses to recognize the cancer as a foreign invader [4]. Therefore, one of the critical steps to overcoming immune tolerance is to find tumor antigens with high immunogenicity as well as wide expression of host-specificity [5].

The antigens that have been used in clinical trials include shared tumor peptides or proteins, heat-shock proteins, tumor cells, and apoptotic bodies [6]. Of these, the shared antigens, such as tyrosinase and melan-A in the case of melanoma, are well-defined and have broad specificity for melanoma patients [7]. However, vaccination with these known antigens is not suitable for all melanoma patients, and it often results in poor immunogenicity and low efficacy [8]. To overcome these limitations, whole autologous tumors that contain both well-known and as yet unknown antigens have been suggested as the best source of patient-specific tumor antigens [9]. The use of autologous tumor cells as antigens dates back to the 1950s [10], and has evolved through various modifications, such as irradiation or genetic transduction to generate expression of cytokines, costimulators, and surrogate foreign antigens [11]. However, using whole cells as vaccine antigens raises safety issues and the risk of adverse effects, e.g., autoimmune diseases, due to inappropriate immune responses, especially those triggered by nuclear autoantigens [12]. Therefore, from the clinical perspective, it is important to extract from autologous tumors those antigens that have fewer adverse effects and that stimulate strong anti-tumor immune responses.

Nano-sized vesicles that mimic the size and geometry of viruses have been reported to induce potent immune responses in a vaccination setting [13]. Application of nano-sized vesicular antigens for cancer vaccine has several advantages over the use of whole cells or soluble proteins, such as higher local concentrations of the antigens, efficient uptake by antigen-presenting cells, and efficient entry into the lymphatic drainage system [14], [15]. A potent nano-sized vesicular antigen source is the exosome, which is a naturally secreted nano-sized vesicle with a size of 30–150 nm [16]. Tumor-derived exosomes contain diverse and distinct sets of cellular proteins, including membrane-bound vesicular antigens and tumor-specific antigens [17]. Studies have shown that tumor-derived exosomes efficiently induce anti-tumor responses both *in vitro* and *in vivo* [18], [19]. However, the clinical use of exosomes in cancer vaccines is hindered by the difficulties associated with obtaining cancer cell lines for each patient and the low yield of exosomes per cell in culture [20], [21].

In the present study, we present a tumor cell-derived and nano-sized vesicular antigen complex, termed the nanovesicle (NV). We purified NVs directly from autologous tumor tissues by homogenization and sonication. The NVs were enriched with tumor-specific antigens, while the nuclear proteins were excluded. The autologous tumor-derived NVs combined with adjuvant induced strong immune responses in dendritic cells (DCs) *in vitro* and showed anti-tumor effects *in vivo* against both residual and metastasized tumors. We believe that our system for engineering the production of the NVs from tumors provides a novel avenue of research into cancer treatment.

## Results

### Preparation and characterization of autologous tumor-derived NVs

**Figure 1:**
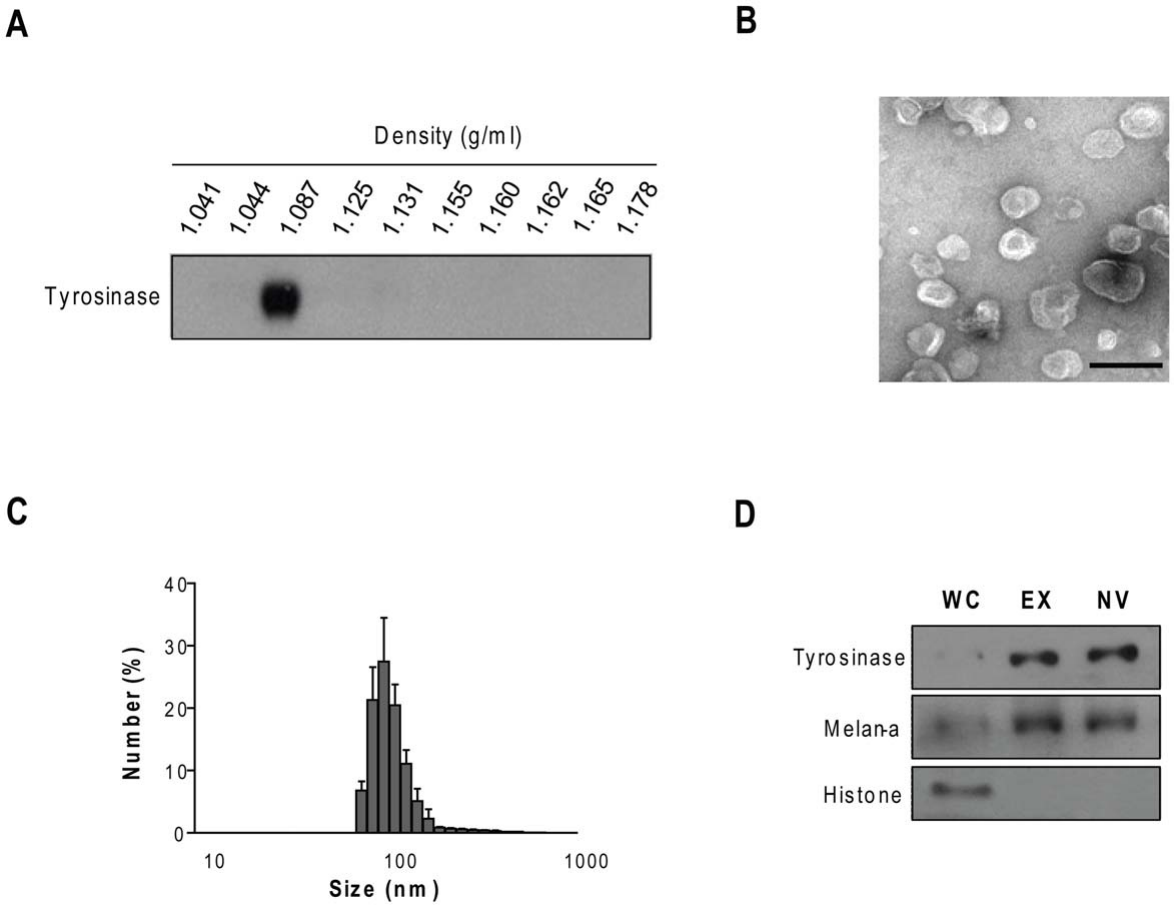
Preparation and characterization of tumor-derived NVs. **A.** Western blot analysis of the melanoma antigen tyrosinase in fractions of tumor-derived NVs obtained from the Optiprep density gradients. The density of each fraction was determined by measuring absorbance at 340 nm. **B.** Transmission electron micrograph of the NVs prepared from autologous melanoma tissues. Bar, 200 nm. **C.** Size distribution of NVs, measured by dynamic light-scattering, shows a diameter range of 80–150 nm. **D.** Western blot detection of tyrosinase, melan-A, and histone in 10 µg of whole cell lysate (WC), exosomes (EX), and NV.

To prepare NVs from autologous tumors, a melanoma tumor was excised from the pre-established melanoma mouse model. The tissue was ground to produce single cells, and disrupted by homogenization. After removal of the nuclei by mild centrifugation, the supernatants were sonicated and re-centrifuged at 10,000×*g* to remove cell debris and mitochondrial fragments. Thereafter, the vesicles were concentrated by sucrose cushion centrifugation. The morphologically intact vesicles were further purified using Optiprep buoyant density centrifugation, to remove protein aggregates and denatured vesicles. The tyrosinase-enriched NVs were floated at a density of 1.087 g/ml (Figure 1A). We obtained 1.8±0.4 mg of NVs per ∼1.5×10^8^ cells of autologous tumor tissue. Transmission electron microscopy of the purified NVs revealed that most of the NVs were small and had the closed vesicular form (Figure 1B). Dynamic light-scattering analysis showed that the mean diameter of the tumor-derived NVs was 101.6±24.8 nm (Figure 1C), which is consistent with the results of a previous study of tumor-derived exosomes [22]. To validate and compare the presence of melanoma antigens in tumor-derived NVs and exosomes, we isolated exosomes from B16BL6 melanoma cells [23]. Western blotting analysis showed that the NVs and exosomes contained similar levels of melanoma antigens, such as tyrosinase and melan-A (Figure 1D). Compared with whole cell lysates, the tumor-derived NVs were enriched for melanoma antigens, but lacked nuclear proteins, e.g., histones (Figure 1D).

### Therapeutic vaccination with the tumor-derived NVs to block tumor growth

**Figure 2:**
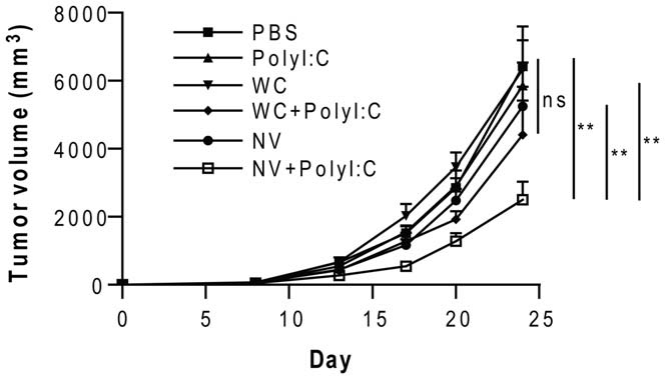
Effect on primary tumor growth of therapeutic vaccination with tumor-derived NVs. Mice (n = 7 per group) that were pre-established with B16BL6 melanoma of the skin received 10 µg of antigens (NVs or whole cell lysates) with or without 50 µg polyI:C per mouse. The mice were subjected to measurements of tumor growth. WC, melanoma whole cell lysates. **P<0.01; ns, not significant.

We assessed the therapeutic effect of the NV vaccine on tumor growth. The tumors were established in C57BL/6 mice by subcutaneous injection of 5×10^5^ B16BL6 cells. When the tumors became visible on Day 7 (volume of ∼50 mm^3^), the mice were vaccinated therapeutically by intraperitoneal injection of the antigens (NVs or whole cell lysates) with or without polyriboinosinic:polyribocytidylic acid (polyI:C). The mice were further vaccinated on Days 14 and 21. Tumor growth was monitored and measured every two to three days. Figure 2 shows that melanoma growth was significantly lower in the group of mice immunized with NVs plus polyI:C than in the PBS-treated (*P*<0.01), NV-treated (*P*<0.01) or polyI:C-treated (*P*<0.01) groups. The T/C ratio (see *Materials and methods*) was also significant based on the National Cancer Institute standard (<42%) in the group that received NVs plus polyI:C (T/C ratio, 34%). However, we found that using tumor whole cell lysates as antigens (WC plus polyI:C) did not induce a significant suppressive effect on tumor growth (*P*>0.05). Collectively, these results indicate that tumor-derived NVs used as antigen-enriched complexes confer efficient therapeutic anti-tumor effects *in vivo*.

### Immune responses induced by the tumor-derived NVs

**Figure 3:**
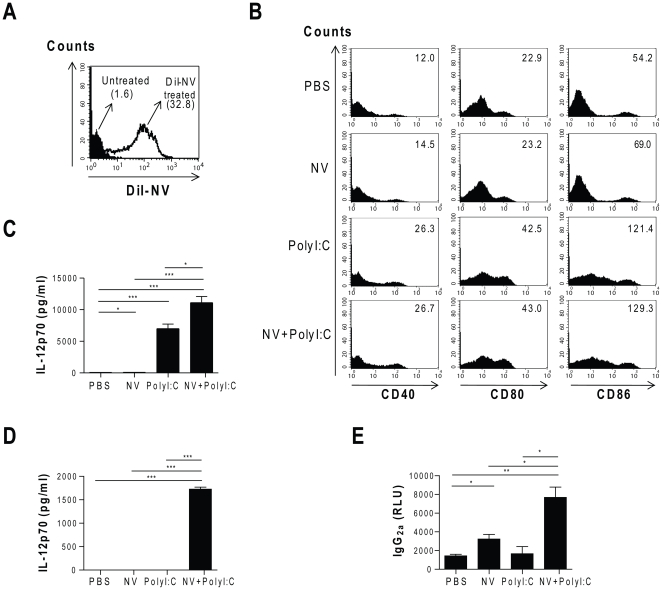
Immune responses induced by tumor-derived NVs. **A.** C57BL/6 mouse-derived bone marrow DCs efficiently take up DiI-labeled NVs (10 µg/ml), as assessed by FACS analysis. The numbers indicate mean fluorescence intensities. **B.** FACS analysis reveals that DCs upregulate the expression of maturation markers CD40, CD80, and CD86 after 24 h of incubation with NVs (2 µg/ml) and polyI:C (10 µg/ml). The numbers in the panels indicate mean fluorescence intensities. **C.** IL-12p70 levels in the supernatants of the cultured DCs, as assessed by ELISA. **D.** Splenocytes were collected from the mice immunized with NVs (10 µg) and/or polyI:C (50 µg), and the levels of secreted IL-12p70 were determined after *in vitro* re-stimulation with NVs. **E.** Serum samples were collected and analyzed by ELISA for the presence of anti-tumor IgG_2a_. *P<0.05; **P<0.01; ***P<0.001.

We verified the role of DCs in the induction of immune responses to melanoma-derived NVs. Bone marrow-derived DCs from the mice were treated with 10 µg/ml of dioctadecyl-tetramethylindocarbocyanine perchlorate (DiI)-labeled NVs for 24 h. FACS analysis revealed the uptake of tumor-derived NVs by DCs (Figure 3A). Moreover, incubation with NVs plus polyI:C increased the levels of DC maturation/activation markers (i.e., CD40, CD80, and CD86), as compared with the PBS-treated and NV-treated groups (Figure 3B). Furthermore, DCs treated with NVs plus polyI:C produced high levels of the Th1 cytokine IL-12p70, as compared with PBS (*P*<0.001), NV (*P*<0.001) or polyI:C (*P*<0.05) treatment (Figure 3C). IL-12p70 was upregulated by treatment with polyI:C alone *in vitro* (Figure 3C), as DCs contain Toll-like receptor 3 [24]. To characterize further the anti-tumor adaptive immune responses *in vivo*, splenocytes and serum samples were isolated from the mice that were immunized with the NVs and/or polyI:C once a week for a total of 3 weeks. Figure 3D shows that *in vitro* re-stimulation of the isolated splenocytes with the NVs significantly increased IL-12p70 secretion only in the group immunized with NVs plus polyI:C (*P*<0.001). In contrast, the Th2 cytokine IL-4 was not detected in any of the groups (data not shown). Moreover, when the humoral response was assessed by the measurement of serum antibody levels, immunization of the mice with NVs plus polyI:C increased Th1-associated antibody, IgG_2a_, production as compared with the PBS (*P*<0.01), NV (*P*<0.05), and polyI:C (*P*<0.05) treatments (Figure 3E). These results suggest that vaccination with tumor-derived NVs primes Th1 immune responses to the established melanoma.

### Therapeutic vaccination with the tumor-derived NVs against pulmonary metastases

**Figure 4:**
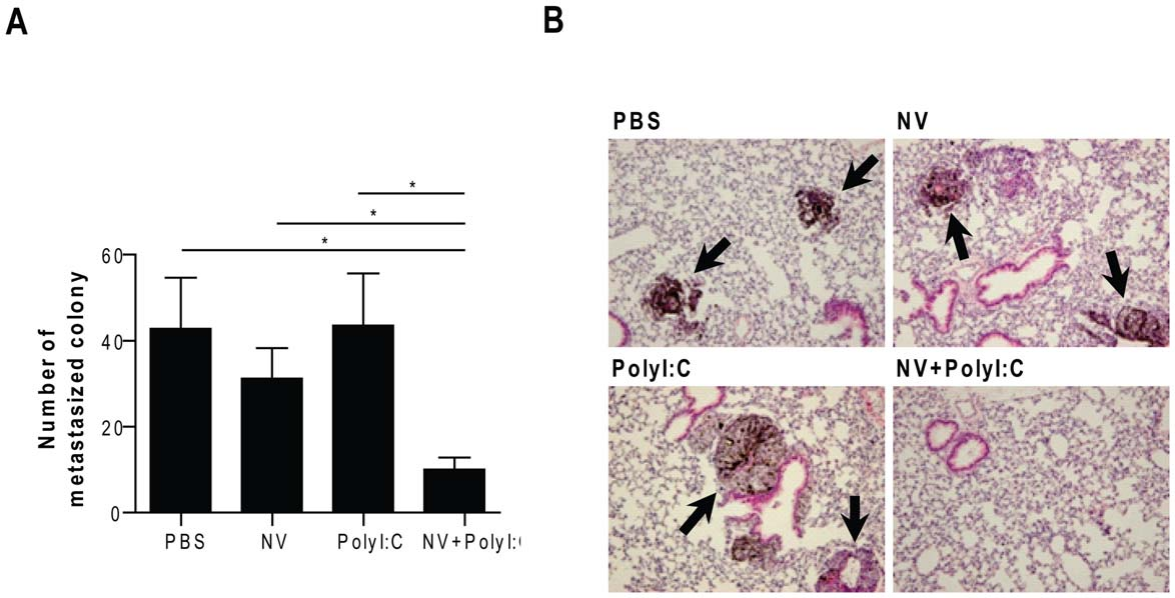
Effect on metastatic tumor growth of therapeutic vaccination with tumor-derived NVs. Mice (n = 5 per group) that were pre-injected intravenously with B16BL6 melanoma cells were administered 10 µg of NVs with or without 50 µg of polyI:C per mouse. **A.** The B16 melanoma pulmonary metastases in the different treatment groups were counted. The data shown are from two independent experiments. *P<0.05. **B.** Histologic sections of representative lung metastases. Arrows indicate the B16 melanoma mass in the lung. Mice immunized with NVs plus polyI:C showed decreases in the number and size of metastasized melanomas. Original magnification ×200; H&E staining.

We investigated the effect of vaccination with the autologous tumor-derived NVs on the growth of metastasized tumors. The pulmonary metastasis model was established by intravenous injection into mice of 1×10^5^ B16BL6 cells. On Days 4, 8 and 12 post-injection, the mice were therapeutically immunized with the NVs and/or polyI:C. On Day 14, the mice were euthanized, and the lung surface tumor nodules were enumerated. As shown in Figure 4A, the mice that were vaccinated with NV plus polyI:C showed reduced numbers of metastasized lung colonies, as compared with the PBS-treated (*P*<0.05), NV-treated (*P*<0.05), and polyI:C-treated (*P*<0.05) groups. Histological analyses also showed that the number and size of the metastasized colonies in the mice immunized with NV plus polyI:C were lower than those in PBS-treated, NV-treated, and polyI:C-treated mice (Figure 4B).

## Discussion

Vaccination with autologous tumor-derived NVs exploits the advantages of autologous tumors as an optimal source of tumor-specific antigens. By deriving NVs directly from tumor tissues, we increased the total yield of vesicular antigens more than 30- to 40-fold, as compared with naturally produced exosomes, without the need for cell line construction from tumor tissues. We obtained about 2 mg of NVs from 1.5×10^8^ cells, and obtained 50 µg of exosomes from the same number of cells. The purified NVs exerted more anti-tumor effects than whole cell lysates, which contain mixtures of soluble and irregular types of membrane antigens. This result is consistent with previous report that exosome-bound antigens induce more potent antigen-specific, anti-tumor immune responses than the soluble forms of the antigens [25]. Therefore, it is important to manipulate the mode of antigen secretion into a vesicular form for vaccination. Thus, tumor-derived NVs may represent a valuable alternative to exosomes. However, it will be necessary to compare the anti-tumor effects of the tumor-derived NVs and exosomes in the near future.

Improving the efficacy of the tumor-derived NV vaccine requires the incorporation of an appropriate adjuvant [6]. To elicit immunity against a syngeneic tumor, we used the Toll-like receptor 3 agonist polyI:C as an adjuvant. We observed that administration of the polyI:C with the tumor-derived NVs delayed tumor growth synergistically and inhibited the B16 lung tumor nodules in the therapeutic vaccination setting. Moreover, NVs complexed with polyI:C induced DC maturation and activation *in vitro* to produce IL-12, which can activate natural killer cells and mediate Th1 immune responses [26]. Further studies are needed to examine in greater detail the anti-tumor mechanisms of this vaccine and whether T cell-mediated immune responses and natural killer cells are involved in blocking tumor growth and metastasis.

Vaccination with tumor-specific antigens and effective adjuvants is a promising treatment option [6], although optimization of tumor NV-based vaccines remains challenging. One way to improve the efficacy of the NV-based vaccine is to use DCs [27]. DCs, which take up tumor-derived exosomes, can induce potent anti-tumor effects on established mouse tumors [16]. Moreover, exosomes or sonicates derived from tumor peptide-loaded DCs have been shown to promote effective anti-tumor effects [28], [29]. As an extension to these studies, the abilities of derivative vesicles from DCs pulsed with the tumor-derived NVs to induce potent anti-tumor immune responses could be studied. Therefore, the efficacy of our NV-based system could be improved by adopting the high-level immune potency of DCs.

In conclusion, the autologous tumor-derived NVs described herein represent a novel source of cell-free cancer antigens. Vaccination with the autologous tumor-derived NVs is versatile and can be applied to diverse cancers, including melanoma. Our tumor-derived NV vaccination strategy could be applied to all patients who undergo surgical removal of the primary tumors.

## Materials and Methods

### Mice, cell lines and reagents

Six-week-old female C57BL/6 mice were purchased from Jackson Laboratories (Bar Harbor, ME, USA). The mice were bred in the pathogen-free facility at POSTECH, and all live animal experiments were approved by the POSTECH Ethics Committee (approval no. 2010-01-0006). The B16BL6 melanoma cell line was maintained in Minimum Essential Medium (Invitrogen, Carlsbad, CA, USA) that was supplemented with 10% (v/v) heat-inactivated FBS (Gibco, Grand Island, NY, USA), 100 U/ml penicillin, and 100 µg/ml streptomycin, and grown at 37°C in 5% CO_2_. PolyI:C (Calbiochem, San Diego, CA, USA) was used as the adjuvant.

### Preparation of tumor-derived NVs

Tumors were surgically excised when they were approximately 1 to 2 cm in diameter. The tumor tissues were homogenized into single cells and washed with PBS. The cell pellet (1.0–1.5×10^8^ cells) was then resuspended in 6 ml of hypotonic solution [10 mM Tris-Cl (pH 7.5), 0.5 mM MgCl_2_, protease inhibitor], incubated for 30 min on ice, and homogenized with 100 strokes in a Dounce homogenizer with a tight-fitting pestle (Wheaton, Millville, NJ, USA). Immediately thereafter, 2 ml of restoration buffer [10 mM Tris-Cl (pH 7.5), 0.5 mM MgCl_2_, 600 mM NaCl] was added, to adjust the tonicity. The homogenate was centrifuged at 500×*g* for 10 min to remove unlyzed cells and nuclei. The 7.8 ml of supernatant was collected, and diluted with 2.2 ml of PBS, and then sonicated using a 40 kHz Branson 2510 bath sonifier (Branson, Danbury, CT, USA) for 30 min at 4°C. This suspension was centrifuged at 10,000×*g* for 10 min at 4°C to remove mitochondria, cell debris, and nuclear fragments [29]. The supernatant was loaded onto a sucrose cushion (0.1 ml of 2.0 M sucrose and 1 ml of 0.8 M sucrose) for ultracentrifugation at 100,000×*g* for 2 h at 4°C, to concentrate the vesicle-containing fraction. The pellet was resuspended in 4.8 ml of 30% Optiprep/HEPES (Axis-Shield PoC AS, Oslo, Norway), and applied to the bottom of a step-density gradient [3.0 ml of 20% Optiprep and 2.5 ml of 5% Optiprep in 10 mM HEPES (pH 7.0), 150 mM NaCl]. After centrifugation at 200,000×*g* for 2 h at 4°C, ten fractions of equal volume were collected from the top of the gradient. The protein concentration of each fraction was determined with the Bradford dye assay (Bio-Rad Laboratories, Hercules, CA, USA). The diameter of the tumor-derived NVs was determined by dynamic light-scattering measurements (Zetasizer Nano S; Malvern Instruments, Worcestershire, UK). All fractions were stored at −80°C until use.

### Preparation of B16BL6-derived exosomes

Exosomes were isolated from B16BL6 melanoma cells, as previously described, with minor modifications [23]. Confluent B16BL6 cells were washed with PBS and then grown in Minimum Essential Medium (Invitrogen) that was supplemented with 10% of exosome-depleted FBS [30]. After 24 h of incubation, the supernatant was collected and serially centrifuged at 300×*g* for 10 min, 2000×*g* for 10 min, and 10,000×*g* for 20 min. The pre-cleared supernatant was ultracentrifuged at 100,000×*g* for 2 h. The resulting pellet, which contained exosomes and possible contaminating proteins, was resuspended in 4.8 ml of 30% Optiprep/HEPES, and applied to the bottom of an Optiprep gradient (3.0 ml of 20% and 2.5 ml of 5% Optiprep/HEPES). After the tubes were centrifuged at 200,000×*g* for 2 h, ten fractions of equal volume were collected from the top of the gradient. The purified exosomes floating on fraction 3 (density, 1.092) were collected, and the protein concentration was determined with the Bradford dye assay.

### Preparation of tumor cell lysates

Tumor tissues that were surgically excised from a mouse were ground into single cells and washed with PBS. The tumor cells were immediately processed by freezing in liquid N_2_, followed immediately by thawing at 37°C [31]. This procedure was repeated three times in rapid succession.

### Western blotting

Samples (10 µg) of the whole cell lysates and tumor-derived NVs were separated by SDS-PAGE (10% resolving gel), and transferred to a PVDF membrane. The blocked membrane was probed with the tyrosinase, melan-A (Santa Cruz Biotechnology, Santa Cruz, CA, USA), or anti-histone antibody (Upstate Biotechnology, Lake Placid, NY, USA), followed by HRP-conjugated anti-mouse or anti-rabbit IgG (Santa Cruz Biotechnology), respectively. The immunoreactive bands were visualized with a chemiluminescent substrate.

### Transmission electron microscopy

The purified tumor-derived NVs were applied to 400-mesh copper grids (EMS, Matfield, PA, USA), and negatively stained with 2% uranyl acetate. Electron micrographs were recorded under the JEM1011 microscope (JEOL, Japan) at an acceleration voltage of 100 kV.

### Engraftment of melanoma onto mouse skin and therapeutic vaccination

B16BL6 cells (5×10^5^) in 100 µl of PBS were subcutaneously injected into the shaved skin of mice. Tumor growth was assessed twice weekly by palpation. Tumor volume was measured with a pair of calipers and calculated using the formula: 1/2×(length×width^2^). For therapeutic vaccination, mice were vaccinated intraperitoneally with tumor-derived whole cell lysates (10 µg) or NVs (10 µg) with or without polyI:C (50 µg) every week after a palpable tumor was established on Day 7. Tumor growth inhibition, defined as the ratio of the median tumor volume for the immunized group versus that for the control group, was calculated as the T/C ratio using the formula: [(median tumor volume of treated group)/(median tumor volume of control group)]×100. The effective criterion for the T/C ratio according to the National Cancer Institute standard is less than 42% [32].

### Isolation of bone marrow-derived DCs

Bone marrow-derived DCs were purified from C57BL/6 mice as described previously, with minor modifications [33]. Briefly, bone marrow cells were harvested and cultured in RPMI1640 medium (Invitrogen) that was supplemented with 10% FBS, 100 U/ml penicillin, 100 µg/ml streptomycin, 50 µM 2-mercaptoethanol, and 20 ng/ml GM-CSF (R&D Systems, Minneapolis, MN, USA) at 37°C in 5% CO_2_. Every two days of culture, half of the medium was removed and replaced with fresh complete medium. On Day 7 of culture, non-adherent and loosely adherent cells were harvested and used for the experiments.

### DC uptake experiments

Tumor-derived NVs (50 µg) in 0.2 ml of PBS were incubated with 5 µM of DiI (Molecular Probes, Eugene, OR, USA) for 20 min at room temperature. DiI-labeled NVs (10 µg/ml) were treated to DCs grown in a 6-well plate (2×10^6^ cells/well). After 24 h incubation at 37°C, cells were collected from the plate. The cells were washed twice with 1 ml of FACS buffer (2% FBS and 0.1% sodium azide in PBS), and then resuspended in 0.3 ml of FACS buffer. The uptake of NVs by DCs was assessed by a FACSCalibur flow cytometer using CellQuest software (BD Biosciences, San Jose, CA, USA).

### DC maturation and activation assay

DCs were seeded at a density of 1×10^6^ cells/well in a 24-well plate, and incubated with 2 µg/ml of NVs and/or 10 µg/ml polyI:C. The cells were collected after 24 h and stained with anti-CD40, anti-CD80, and anti-CD86 antibodies (BD Biosciences), to analyze the levels of cell surface markers by flow cytometry. The conditioned media of the cultured cells were harvested for the determination of IL-12p70 production using an ELISA kit (R&D Systems) according to the manufacturer's instruction.

### Measurements of splenocyte cytokine levels and serum antibody levels in mice immunized with tumor-derived NVs

C57BL/6 mice were immunized intraperitoneally on Weeks 0, 1, and 2 with 10 µg of tumor-derived NVs and/or 50 µg polyI:C. The control groups were immunized with PBS. One day after the last immunization, the mice were euthanized. Splenocyte suspensions were prepared and stimulated with 2 µg/ml of NVs. The supernatants were harvested after 72 h, and the levels of cytokines were assayed by ELISA. Serum samples from the mice were used to determine the circulating antibody levels by ELISA. Briefly, flat-bottom 96-well plates were coated overnight with 1 µg of NV, blocked with 1% BSA, and incubated with a 1∶10 dilution of mouse serum for the detection of anti-NV IgG_2a_ antibodies (Bethyl Labs, Montgomery, TX, USA).

### Engraftment of melanoma into the mouse lung and therapeutic vaccination

Engraftment of melanoma into the lungs of mice was performed by intravenous injection of B16BL6 cells (1×10^5^) in 100 µl of PBS. For therapeutic vaccination, the mice were vaccinated intraperitoneally with NVs (10 µg) with or without polyI:C (50 µg) on Days 4, 8, and 12. The melanoma metastases on the surfaces of the lungs were counted 14 days post-challenge. These experiments were carried out with groups of five mice and were repeated twice.

### Histology

Excised tissues were fixed with 4% paraformaldehyde. The tissues were embedded in paraffin and sectioned at 4-µm thickness. The paraffin sections were deparaffinized with xylene and stained with hematoxylin and eosin (H&E). Images were acquired using an Olympus BX51 microscope.

### Statistical analysis

Data are presented as mean values with standard errors. Significant differences between the treatments were assessed using the Student's *t*-test. A *P*-value of <0.05 was considered statistically significant.
